# Phenolic Profile and Susceptibility to *Fusarium* Infection of Pigmented Maize Cultivars

**DOI:** 10.3389/fpls.2018.01189

**Published:** 2018-08-14

**Authors:** Jamila Bernardi, Lorenzo Stagnati, Luigi Lucini, Gabriele Rocchetti, Alessandra Lanubile, Carolina Cortellini, Giovanni De Poli, Matteo Busconi, Adriano Marocco

**Affiliations:** ^1^Department of Sustainable Crop Production, Università Cattolica del Sacro Cuore, Piacenza, Italy; ^2^Department for Sustainable Food Process, Università Cattolica del Sacro Cuore, Piacenza, Italy; ^3^Research Centre for Biodiversity and Ancient DNA, Università Cattolica del Sacro Cuore, Piacenza, Italy; ^4^Department of Animal Science, Food and Nutrition, Università Cattolica del Sacro Cuore, Piacenza, Italy; ^5^Terra Srl, Cremona, Italy

**Keywords:** flavonoids, *Fusarium*, metabolomics, anthocyanins, FER, phytoalexins

## Abstract

Maize is a staple food source in the world, whose ancient varieties or landraces are receiving a growing attention. In this work, two Italian maize cultivars with pigmented kernels and one inbred line were investigated for untargeted phenolic profile, *in vitro* antioxidant capacity and resistance to *Fusarium* *verticillioides* infection. “Rostrato Rosso” was the richest in anthocyanins whilst phenolic acids were the second class in abundance, with comparable values detected between cultivars. Tyrosol equivalents were also the highest in “Rostrato Rosso” (822.4 mg kg^−1^). Coherently, “Rostrato Rosso” was highly resistant to fungal penetration and diffusion. These preliminary findings might help in breeding programs, aiming to develop maize lines more resistant to infections and with improved nutraceutical value.

## Introduction

Maize is the most cultivated cereal grain throughout the world, considering both yield and harvested area (Organization for Economic Cooperation and Development-OECD and FAO, 2015). Maize is a staple crop in the African region and South America, while in developed countries is used mainly to feed livestock as forage, silage and grain rather than as biofuel and for industrial uses. A new study by FAO and OECD estimates that global consumption of cereals will increase by 390 million tons between 2014 and 2024. The core driver of the increase will be the rising demand for animal feed, of which about 70% is maize, accounting for more than half of the total (FAO, 2014).

Endosperm is the main storage tissue in maize kernel, accumulating carbohydrates, such as starch (90–95%), and storage proteins, such as prolamins (10–12%) (Wu and Messing, 2014). Considering its nutritional value, other important components in maize kernels are: carotenoids, flavonoids and hydroxycinnamic acids. Carotenoids are the common pigments in maize, insoluble compounds that accumulate in the endosperm and confer the typical orange color. Most of the cultivated maize has yellow kernels, but some varieties possess the ability to pigment different tissues (i.e., anthers and roots), especially in response to stresses. Phenolic compounds such as flavonoids, are responsible for the red, purple, blue and black coloration of kernels and other parts of the plant. In maize seed, accumulation of pigments can occur in two tissues, namely the pericarp, the maternal-derived tissue, rather than the aleurone that is the peripheral part of the endosperm. Anthocyanins are water-soluble flavonoids that accumulate in vacuoles of the aleurone. Other colored flavonoids in maize are the red pigments phlobaphenes, polymers of the flavan-4-ols apiforol and luteoforol that accumulate in the seed pericarp and cob glumes of maize (Sharma et al., 2012). Flavonoids, like other phenolics, have the power to protect the kernel from biotic and abiotic stresses, and have been associated with the beneficial effects of the Mediterranean diet, given their potent antiangiogenic, anti-inflammatory, and anticarcinogenic activities (Petroni and Tonelli, 2011). In rats fed with anthocyanin-rich maize, the amount of cardiac tissue that was damaged following ischemic conditions was reduced by approximately 30% compared to rats fed with anthocyanin-free maize (Toufektsian et al., 2011). Anthocyanins from purple corn also prevent weight gain and obesity in mice under high fat diet, and they can reduce severe diabetic complications (Tsuda, 2012). Phenolic compounds that accumulate in the maize endosperm and pericarp may contribute to resistance against insect damages, Fusarium ear rot (FER) and fumonisin contamination (Sharma et al., 2012; Atanasova-Penichon et al., 2016). Indeed, flavonoids could act as physical impediment against fungal attack (in particular when accumulated in the pericarp) by hardening maize kernel thus reducing the spread of mycelium in the inner parts of the seeds (Atanasova-Penichon et al., 2016). Other flavonoids are known to reduce insect attack like the flavone maysin (a *C*-glycosyl luteolin derivative) that can decrease the growth of earworm larvae in maize (Sharma et al., 2012).

In the last years, there was a growing demand of consumers in increasing the phenolic content of vegetables by rediscovering ancient cultivars that possess a natural pigmentation, because they are expected to have higher nutritional value (Falcone Ferreyra et al., 2012; Casas et al., 2014). With this regard, metabolomics has been proposed as a powerful tool to achieve a comprehensive picture of the phenolic signature in crop foods (Rocchetti et al., 2017, 2018a). Nonetheless, the actual phenolic profile is also supposed to play a range of physiological roles in plant, including protection toward both abiotic (e.g., UV radiation or oxidative stress via radical scavenging) and biotic stresses (Nakabayashi and Saito, 2015; Talhaoui et al., 2015). Extensive literature can be found on phenolics in non-pigmented maize, whereas most of the work on pigmented maize referred to blue genotypes from Mexico. However, red-purple genotypes received limited attention to date, although they have been recently reported to possess a favorable nutritional profile (Rocchetti et al., 2018b). Furthermore, previous works investigated phenolic profile of maize through targeted approaches, and therefore they might have not comprehensively screened the whole profile, including eventual conjugates and glycosylated compounds.

Therefore, the aim of this work was to study some maize genotypes featured by red-purple kernel pigmentation, according to their field performance, phenolic profile, *in vitro* antioxidant capacity and resistance to *Fusarium* infection. Assumed that phenolics alone are not the unique component in the resistance to *Fusarium* infection, it becomes relevant to test to which extent these compounds can interfere with fungal spread. On these bases, the phenolic profiles and resistance to *Fusarium* of three genotypes were compared to a non-pigmented commercial maize hybrid. In fact, these genotypes received less attention than other pigmented maize genotypes, even they might have a double attitude both in disease resistance and functional ingredients. A recent study on starch digestibility after cooking highlighted that the three genotypes “Nostrano della Val di Non” and “Rostrato Rosso,” and a “Purple B73” possessed distinctive and diverse pigmentation patterns that could be linked to the modulation of starch digestibility (Rocchetti et al., 2018b). On these bases, the ultimate aim of this work was to investigate the potential exploitation of the above-mentioned cultivars in breeding programs rather than gaining information on their viability as functional foods.

## Materials and Methods

### Maize Germplasm

**FIGURE 1:**
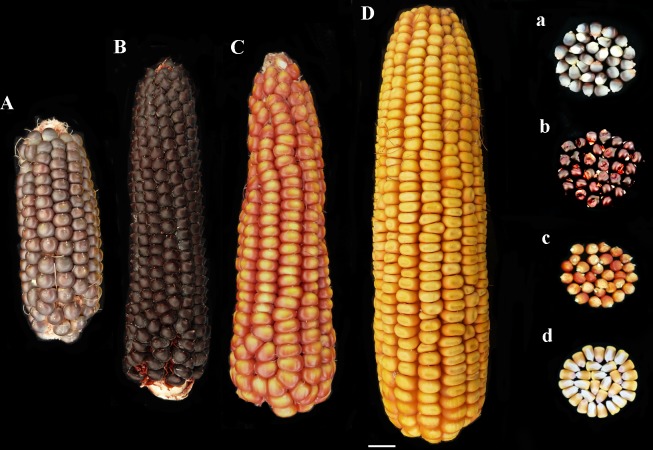
Phenotype of ears and kernels of “Purple B73” **(Aa)**, “Rostrato Rosso” **(Bb)**, “Nostrano della Val di Non” **(Cc)**, and control **(Dd)**.

Two pigmented cultivars “Nostrano della Val di Non” and “Rostrato Rosso”, and a “Purple B73” line, were provided by ISTA, Agroalimentare Sud S.p.A. (Lodi, Italy). The yellow maize hybrid Agrister (Limagrain, Saint-Beauzire- France) and the B73 inbred line (available as our stock) were used as non-pigmented control (**Figures 1Aa–Dd**). The former non-pigmented genotype was used as reference for field evaluation, phenolic profile and antioxidant capacity investigations. However, the latter was a fungicide-free inbred line to be used as control for both *in vitro* and field infection assays.

### Field Evaluation

The maize genotypes were sown on April 2015 in randomized blocks, with four replicates per genotype, including the commercial hybrid Agrister, in the experimental field located in San Damiano (PC), Italy (44°54′10.7″N, 9°41′25.6″E). Plots consisted of six rows 5 m long and spaced 0.75 m apart, planting 25 seeds per row. Each plot was spaced apart by four rows of the commercial hybrid Agrister and by a 5 m block of hybrid on the edge. Standard agricultural practices were followed. Phenotypic evaluation was performed according to the UPOV characters (ASFIS, 1992). Ears were harvested at maturity, kernels were sampled and kept at −20°C for further analyses on phenolic profile and antioxidant capacity.

A further experiment was carried out on April 2017, using the same genotypes, to investigate resistance to *Fusarium*. The field trials were located at CERZOO “Centro Di Ricerche Per La Zootecnia E L′Ambiente S.C.R.L.” facilities (San Bonico 45.003624N, 9.705179E, Piacenza, Italy). Plot scheme and agricultural practices were the same as for previous trials. The “Rostrato Rosso” and “Nostrano della Val di Non” plants derived from a second-cycle of selfing.

### Resistance to Artificial Infection

In the field trial, plants were hand self-pollinated and at 15 days after pollination ears were artificially inoculated with a spore suspension of *F. verticillioides* ITEM10027 (MPVP 294, 1 × 10^6^ spores per mL) according to the pin-bar method (Maschietto et al., 2017). Ears were harvested at maturity and phenotypically evaluated for FER severity using a seven-point scale (Maschietto et al., 2017). Ears not inoculated were harvested at maturity and kernels used for the further investigation through *in vitro* infection, according to the rolled towel assay (RTA) (Lanubile et al., 2015). With this aim, seeds with similar size and shape, preferably flattened and without visible damage, were selected from each maize genotype. To minimize at most the presence of contaminating fungi, the seeds were surface-sterilized by shaking them in 50-mL tubes at room temperature with 70% ethanol for 5 min, washed by sterilized bi-distilled water for 1 min, then with commercial bleach solution for 10 min, and finally rinsed three times with sterile distilled water for 5 min each time (Lanubile et al., 2015). Two towels of germination paper (Anchor Paper, Saint Paul, MN, United States) were moistened with sterilized distilled water. For each genotype, 20 seeds were placed on the germination paper about 10 cm from the top of the towel with the embryo side facing out. Kernels were inoculated on the embryo side near the pedicel with 100 μL of a 1 × 10^6^ spores per ml suspension of *F. verticillioides* ITEM10027 (MPVP 294) and covered with another moistened towel. Towels were rolled up and arranged vertically in a 25-L bucket covered with a black plastic bag and kept for 7 days at 25°C in the dark. For each genotype, a control RTA was prepared as previously described, but avoiding the inoculation step.

Seedlings were rated using a five-point severity scale adapted from previous research on soybean seedlings (Lanubile et al., 2015). According to this scale, the scores were as follows: 1 = healthy, germinated seedlings with no visible signs of colonization; 2 = germination and colonization of the kernel near the pedicel; 3 = germination with widespread colonization of the kernel and browning of the coleoptile; 4 = germination with reduced seedling development, complete colonization of the kernel, and lesions and abundant mold on the shoot, 5 = no germination due to complete rotting of the kernel. Severity after inoculation (SEV_I) and in the control assays (SEV_C) were recorded for each genotype.

### Extraction of Phenolic Compounds

The maize samples were randomly collected from four ears within each plot of the same genotype, obtaining six replicates. Kernels were grinded in a laboratory mill equipped with a 1-mm screen. Thereafter, 10 ml of a hydro-alcoholic solution (80% methanol, acidified with 0.1% formic acid, v/v) was used to extract phenolic compounds from 1 g of each replicate, by means of an Ultra-Turrax (25,000 rpm for 3 min). Suspensions were centrifuged 6,000 ×*g*, and then 5% trichoroacetic acid (TCA) was added to the liquid phase to precipitate proteins. The solutions were stored overnight in freezer, at −18°C, filtered by means of a 0.22 μm syringe cellulose filter, and stored in dark vials at −18°C until further analyses. Six individual replicates were extracted from each genotype.

### UHPLC-ESI/QTOF Screening of Phenolic Compounds

The comprehensive phenolic profile of maize samples was investigated through an untargeted metabolomic approach, using ultra-high-pressure liquid chromatography (UHPLC) in combination to a quadrupole-time-of-flight (QTOF) mass spectrometer. In more detail, the instrumentation consisted of a 1290 UHPLC coupled to a G6550 mass spectrometer (all from Agilent Technologies, Santa Clara, CA, United States) via a JetStream dual electrospray ionization source. Chromatographic and mass spectrometric instrumental conditions to screen phenolic compounds in the samples were optimized in previous works (Borgognone et al., 2016; Rocchetti et al., 2017). Briefly, a Knauer BlueOrchid C18 column (100 mm × 2 mm, 1.8 μm) was used for chromatographic separation, using a binary mixture of methanol and water as mobile phase (LCMS grade, VWR, Milan, Italy). The gradient elution was designed to increase methanol from 5 to 95% within 34 min with a flow rate of 220 μL min^−1^ and a 3.5 μL injection volume. The mass spectrometer was set up to positive SCAN mode, detecting masses in the range 100–1000 m/z. Each extract was injected once, as single instrumental replicate.

The software Profinder B.07 (from Agilent Technologies) was used to elaborate raw data, and polyphenols annotation was carried out using the database Phenol-Explorer 3.6 (Rothwell et al., 2013), considering the whole isotopic pattern. The “find-by-formula” algorithm, that includes monoisotopic mass, isotopes spacing and ratio, was used for annotation (mass accuracy tolerance < 5 ppm). Thereafter, mass and retention time alignment and filter-by-frequency (features not present in at least one treatment in 100% of replications were discarded) were applied.

In order to provide quantitative information, phenolic compounds were firstly ascribed into classes and sub-classes, and then cumulative intensity for each phenolic sub-class were converted in mg kg^−1^ equivalents, by means of calibration curves from nine polyphenol standards (from Extrasynthese, Lyon, France). Furofuran lignans were quantified as sesamin, dibenzylbutyrolactone lignans as matairesinol, phenolic acids as ferulic acid, anthocyanins as cyanidin, tyrosols and low-molecular-weight phenolics as tyrosol, alkylresorcinols as 5-pentadecylresorcinol (also known as cardol), stilbenes as resveratrol, flavanols and flavonols as catechin, and flavones as luteolin equivalents. Calibration curves were built using a linear fitting (un-weighted and not forced to axis-origin) in the range 0.05–500 mg L^−1^; a coefficient of determination *R*^2^ > 0.97 was used as acceptability threshold for calibration purposes.

### *In vitro* Antioxidant Capacity Assays

Antioxidant capacity assays were carried out on the same extracts used for phenolic profiling. The *in vitro* antioxidant capacity of each maize sample was evaluated as radical scavenging ability (DPPH assay) and ferric reducing power (FRAP assay), as previously described (Ghisoni et al., 2017). Briefly, 1.5 mL of maize extract was combined to the same volume of an ethanol solution of DPPH (1.0 mM). The absorbance was recorded at 5-min intervals (until the steady state) to 517 nm using a Perkin Elmer Lambda 12 spectrophotometer (Ontario, Canada). The results were finally expressed as gallic acid equivalents (GAE).

The FRAP antioxidant assay was carried out by means of a clinical analyzer ILAB 600 (Instrumentation Laboratory, Lexington, MA, United States). The FRAP working reagent consist of acetate buffer (300 mM, pH 3.6), TPTZ (10 mM) in 40 mM HCl and FeCl3 (20 mM), in the ratio 10:1:1 (v/v). Each extract (100 μL) was combined to 3 ml of FRAP working reagent, and the absorbance was recorded at λ = 600 nm, after 243 s of incubation at 37°C. The FRAP results were expressed as GAE.

### Statistical Analysis

The analysis of variance for the agronomic traits and the statistical analysis of artificially infected samples was performed using the R software. Phenotypic values collected in the RTA experiment were square-root transformed and mean values of severity were calculated. FER phenotypes, scored as percentages, were arccosine transformed before performing statistical analysis. The Kruskal–Wallis one-way analysis of variance and the Kruskal–Dunn *post hoc* test, available in the R package PMCMR (Pohlert, 2014) were applied to detect significant differences between maize accessions tested in the RTA experiment; the Welch one-way ANOVA and the Games–Howell *post hoc* test, available in the R packages one waytes (Dag et al., 2017) and user friendly science (Peters, 2017) were applied for FER field evaluation.

Analysis of variance for *in vitro* antioxidant capacity (one-way ANOVA, *P* < 0.05) and correlations between antioxidant capacity and concentration of different phenolic classes (Pearson, two-tails) were carried out in IBM SPSS statistics 24.

Metabolomics data were elaborated by means of Agilent Mass Profiler Professional B.12.06 software, as previously described (Rocchetti et al., 2017). Abundance of phenolic compounds was normalized at the 75th percentile and corrected for the corresponding median in all samples. Volcano plots, carried out combining ANOVA (*p* < 0.01, Bonferroni multiple testing correction) and fold-change (FC) analysis (cut-off = 5), and Venn diagrams were also generated. The not-supervised statistical approach Hierarchical Cluster Analysis was then carried out as previously described (Rocchetti et al., 2017). Finally, the raw data were elaborated into SIMCA 13 (Umetrics, Malmo, Sweden) for the supervised statistical approach orthogonal projection to latent structures discriminant analysis (OPLS-DA). A confidence limit of 95 and 99% was used to investigate the presence of outliers in the model (according to Hotelling’s T2 approach), while cross validation (CV-ANOVA, *p* < 0.01) together with a permutation test (*N* = 100) to exclude overfitting, were then carried out. The goodness-of-fit and prediction ability of the model (i.e., *R*^2^Y and *Q*^2^Y, respectively) were also investigated, adopting cut-off values provided in literature (Rombouts et al., 2017). Finally, the VIP analysis was carried out to investigate the variable’s importance in projection, i.e., considering those phenolic metabolites with the highest discrimination potential (VIP score > 1.2).

## Results

### Phenotyping of Pigmented Maize Genotypes

Three colored genotypes (“Nostrano della Val di Non,” “Rostrato Rosso” and “Purple B73”) and a yellow hybrid used as reference (“Agrister”), were characterized both for vegetative and reproductive traits. Agronomic measurements of the main traits are summarized in **Table 1** whereas all the parameters are listed in **Table 2**.

**Table 1:** Main agronomic traits of the four maize genotypes.

Agronomic traits	Agrister	Val di Non	Purple B73	Rostrato Rosso
Plant height (cm)	190–200^d^	130–170^b^	140–150^a^	170–180^c^
Ear height (cm)	80–90^c^	70–80^a^	70–80^a^	100–110^b^
Number of rows per ear	18–20^a^	10–14^b^	16^a^	12–14^c^
Number of plants per plot	116.2^c^	105.5^b^	93.5^a^	95.2^a^
Number of ears per plot	111.7^c^	43.2^a^	63.2^b^	54.5^ab^
Grain weight per plot (g)	13660.6^a^	1172.1^b^	1032.9^b^	1247.3^b^
% of barren plants	1.5^c^	62.2^b^	30.2^a^	40.7^a^

*Within the same column, means followed by the same letter are not significantly different at P ≤ 0.05 according to Fisher’s test.*

**Table 2:** Morphological and physiological traits of all the genotypes according to the UPOV characters.

Trait	VdN^a^	RR^b^	Purple B73	Agrister
First leaf: anthocyanin coloration of sheath	1	1	3	1
Leaf: angle between leaf and stem	1	3	1	1
Leaf: attitude of leaf	1–3	3	1	1
Stem: zig-zag attitude	1	5	2	1
Stem: anthocyanin coloration of secondary roots	5	5	3	3
Tassel: time of male flowering (days after sowing)	20–06	30–06	08–07	28–07
Tassel: time of male flowering (GDD)	535	636	775	1063
Tassel: anthocyanin coloration of ring of the glume	3–5	3	3	1
Tassel: anthocyanin coloration of the glumes	3–5	1	9	1
Tassel: anthocyanin coloration of the anthers	3	3	1	5
Tassel: density of main axis	5	3	5	5
Tassel: angle between main axis and lateral branches	1	1–7	3	1
Tassel: attitude of lateral branches	3	1–7	1	5
Tassel: number of lateral branches	7–9	7–9	3-5	5–6
Ear: silking time (days after sowing)	23–06	04–07	10–07	01–08
Ear: silking time (GDD)	535	109	804	1120
Ear: anthocyanin coloration of the silks	1	1	1	1
Leaf: anthocyanin coloration of sheat	1–3	1–3	9	1
Tassel: length of main axis above lowest side branch	5	5–7	3	7
Tassel: length of main axis above highest side branch	5	3–5	3	7
Tassel: length of lowest lateral branch	1	1–3	1	7
Plant: height (upper-leaf)	130–170	170–180	140–150	190–200
Plant: ear height (upper-ear)	70–80	100–110	70–80	80–90
Plant: height of ear relative to plant length	5	9	7	3
Leaf: width of blade	1	1	1	1
Ear: length of peduncle	3	7	3	3
Ear: length of ear	5	9	3	9
Ear: diameter of ear	3	3	3	7
Ear: shape of ear	2	2	2	2
Ear: number of rows	3	3	5	7
Ear: type of grain	2	3	2	5
Ear: color of the tip of grain	6	9	8	3
Ear: color of the dorsal side of grain	4	9	1	1
Ear: anthocyanin pigmentation of the glumes of cob	5	9	7	3
Physiological maturity (days after sowing)	10–08	15–08	18–08	15–09
Physiological maturity (GDD)	1234	1291	1327	1666
Stem: anthocyanin pigmentation of nodes	1	7	7	3
Stem: anthocyanin pigmentation of internodes	1	5	5	3

*^a^VdN, Nostrano della Val di Non; ^b^RR, Rostrato Rosso.*

#### “Purple B73”

“Purple B73” is a medium-short inbred line; given this, plants are phenotypically uniform, with a very strong purple pigmentation of the stalk, leaves, glumes, bracts and tassel while silks and anthers are white (**Table 2**). Ears are classified as short of 15–18 cm with violet – deep-gray kernels arranged in 16 rows (**Figure 1Aa**). This inbred line reached silking at 804 GDD and physiological maturity at 1327 GDD (**Table 2**). The purple corn is resistant to stalk lodging and the percentage of barren plants was around 30%.

#### “Rostrato Rosso”

The “Rostrato Rosso” plants are medium-high tall with a short cycle, silking was at 709 GDD and physiological maturity at 1291 GDD. The variety is variable for anthocyanin pigmentation of the leaf sheath that can be deep green or with purple strikes, also the tassel attitude may vary between erect or pendulous (**Table 2**). Ears are morphologically uniform with flint violet–black kernels with a pronounced rostrum (**Figure 1Bb**). The ear is slightly conical and longer than 20 cm, kernels are arranged in 12–14 rows. Forty-one percent of plants were earless and lodging was noted for this variety. Lodging can be a consequence of the very high ratio of ear insertion related to plant height (**Table 2**).

#### “Nostrano della Val di Non”

The “Nostrano della Val di Non,” hereafter called “Val di Non” has a low-medium height and reached mid silk 1 week before the other genotypes getting the physiological maturity at 1234 growing-degree days (GDD; **Table 2**). Neither leaves nor the stem are pigmented while silks and kernels are pigmented (**Table 2**). The kernel pigmentation can vary from deep orange to dark red (**Figure 1Cc**). Variability has been observed in the anthocyanin pigmentation of tassel glumes that could be violet, red or intermediate colors. Nonetheless the number of barren plants was quite high (62%) the production was good since this plant did not present lodging symptoms (**Table 1**).

### Phenolic Profile of Pigmented and Yellow Genotypes

The phenolic profile in pigmented genotypes (i.e., “Purple B73,” “Rostrato Rosso,” and “Val di Non”) and in non-pigmented references (i.e., “Agrister” and “B73 line”) was investigated using an untargeted metabolomics approach based on UHPLC-ESI/QTOF mass spectrometry. Overall, phenolic profile was diverse, with flavonoids being the most frequently detected class detected (152 annotated compounds: 46 anthocyanins, 48 flavanols and 58 flavones), followed by phenolic acids (55 compounds), tyrosols (43 compounds), alkylphenols (14 compounds) and other phenolics (20 lignans and 6 stilbenes). All the comprehensive information regarding phenolic compounds identified across the different maize samples are provided as supporting information (**Supplementary Tables S2**, **S3**), including annotations (raw formula, identification scores) and composite spectra (masses and abundances).

**FIGURE 2:**
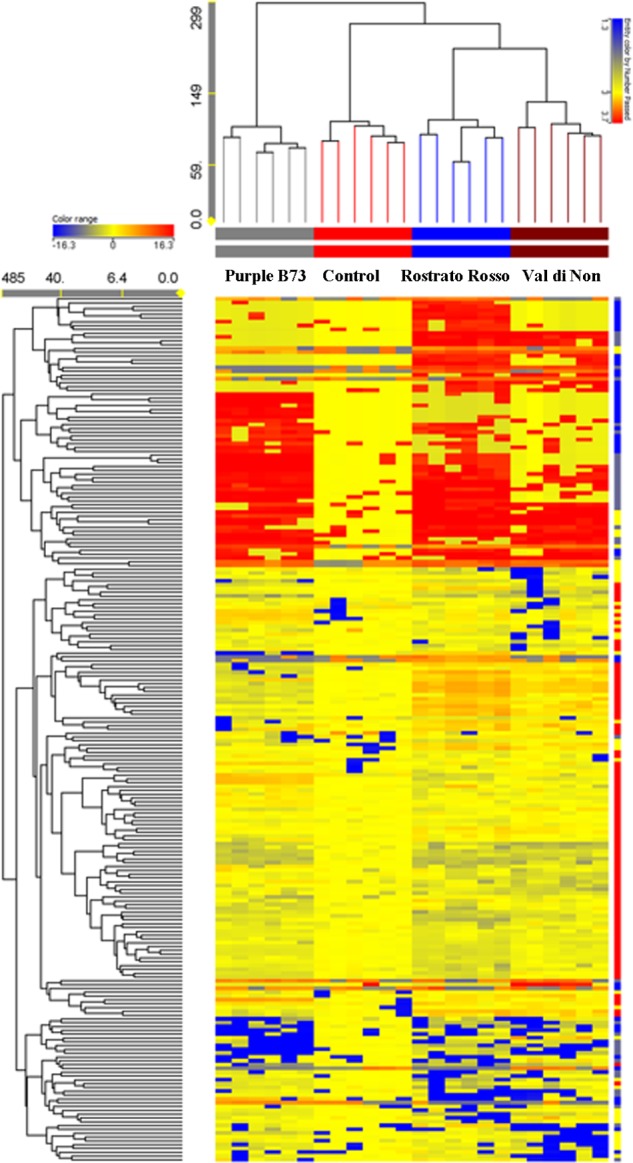
Not averaged unsupervised hierarchical clustering on the phenolic profile of maize samples (similarity: Euclidean; linkage rule: Ward). Compounds’ intensity was used to build up fold-change based heat map, on the bases of which clusters analysis was done. Agrister was used as a non-pigmented kernel control.

The Agrister yellow maize was used as control for both phenolic profiling and related antioxidant capacity, since this former was grown together with pigmented genotypes, i.e., under the same pedoclimatic conditions. Unsupervised hierarchical cluster analysis was then produced considering the fold-change heat map, highlighting a substantial change of the phenolic profile moving from the Agrister yellow maize (control) toward the pigmented genotypes (**Figure 2**). The output of the heat map showed two main clusters; the first one was represented by the line “Purple B73,” while the second cluster included “Rostrato Rosso” and “Val di Non,” together with the control. However, this latter showed a distinct phenolic profile, when compared to pigmented varieties, being in a separate sub-cluster. Nevertheless, the heat map of the hierarchical cluster analysis clearly showed that the abundance of several phenolics tends to disappear moving from yellow to pigmented varieties.

**FIGURE 3:**
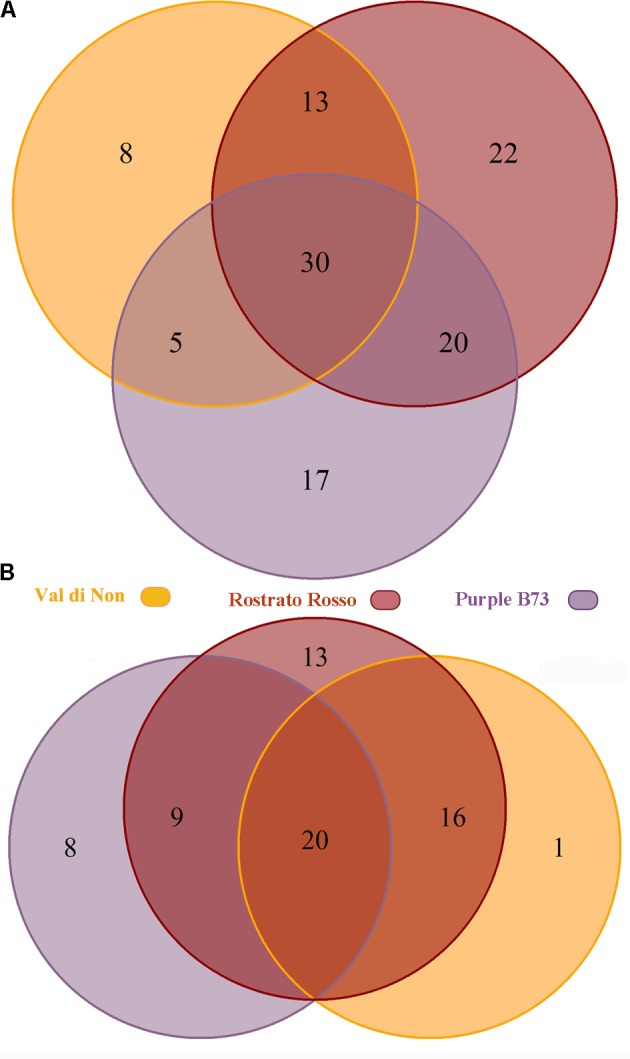
Venn diagram of differentially accumulated compounds (Volcano plot analysis, *P* < 0.01, fold-change > 5), comparing each of the three genotypes against Agrister. The upper diagram **(A)** represents all the differential phenolic compounds, while the lower one **(B)** only the up-regulated compounds.

Subsequently, the Venn diagrams were carried out in order to shed light on differentially and common phenolic compounds, considering pigmented varieties and Agrister yellow maize (**Figure 3**). Overall, the output of Venn diagrams showed that the three pigmented genotypes had 30 common phenolic compounds. Nevertheless, “Val di Non” possessed 8 exclusive compounds (**Supplementary Table S1**), being above all phenolic acids (coumaric acid, coumaric acid 4-*O*-glucoside, and hydroxycaffeic acid), while “Rostrato Rosso” and “Purple B73” reported 22 and 17 exclusive phenolics, respectively. Interestingly, among phenolic compounds characterizing the “Rostrato Rosso,” several cyanidin-derivatives forms were detected, i.e., pelargonidin 3-*O*-glucosyl-rutinoside/sophoroside, cyanidin 3,5-*O*-diglucoside and cyanidin 3-*O*-sambubioside, delphinidin 3-*O*-xyloside, and malvidin 3-*O*-(6″-acetyl-galactoside). “Purple B73” showed an abundance of flavonoids as exclusive compounds, being characterized by flavones, flavonols and anthocyanins.

Considering specific compounds, apiforol that is a precursor of phlobaphenes was up accumulated in “Val di Non” and “Rostrato Rosso” respect to both “Purple B73” and yellow maize. This evidence suggests that these two cultivars could accumulate also phlobaphenes in kernels, additionally to the other phenolics detected. Furthermore, the compound maysin was up accumulated in “Rostrato Rosso” respect to other pigmented genotypes.

**FIGURE 4:**
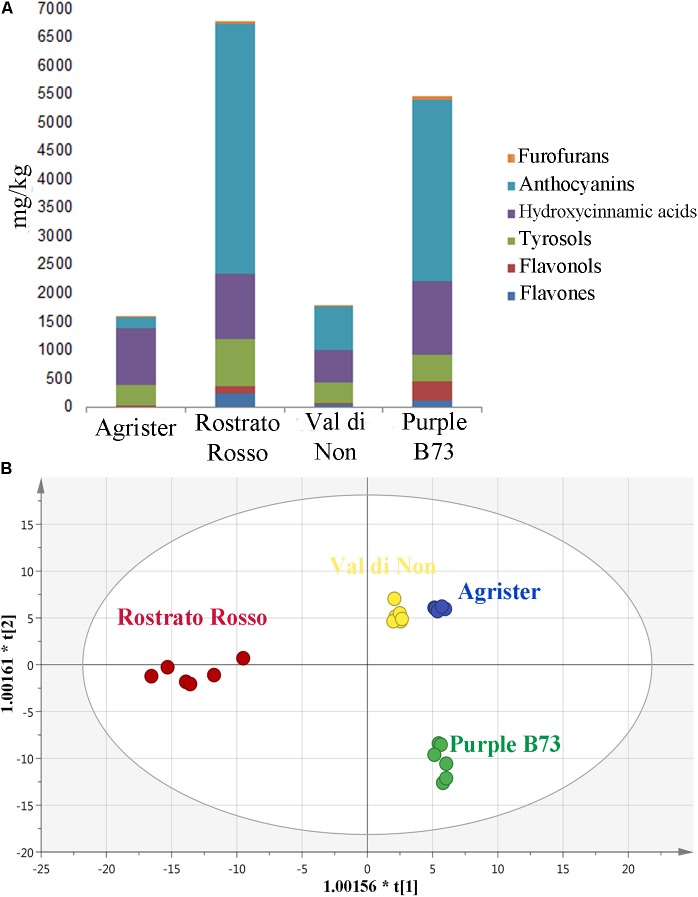
**(A)** Cumulative abundance, expressed as mg kg^−1^ equivalents, of the different phenolic subclasses in the analyzed maize genotypes, as gained from UHPLC-ESI/QTOF-MS screening analysis. **(B)** Orthogonal Projections to Latent Structures Discriminant Analysis (OPLS-DA) on maize genotypes according to their phenolic profile. Individual replications are given in the class prediction model score plot.

Starting from these differences, the relative abundance of each phenolic subclass was investigated according to the curves from the respective phenolic standards (**Figure 4A** and **Table 3**). Remarkably, the flavonoid profile was very explicative, allowing to clearly discriminate among the four maize genotypes; “Rostrato Rosso” and “Purple B73” were the richest in anthocyanins, being 4399.4 and 3167.9 mg kg^−1^, respectively, while “Val di Non” and the yellow maize (used as control) showed the lowest values in anthocyanin equivalents (752.5 and 205.4 mg kg^−1^, respectively). The second phenolic class recorded in abundance was that of phenolic acids, with comparable values detected for “Purple B73” (1305.9 mg kg^−1^), “Rostrato Rosso” (1149.7 mg kg^−1^), and the control sample (979.6 mg kg^−1^), while the lowest values were recorded in “Val di Non” (589.2 mg kg^−1^). Furthermore, “Rostrato Rosso” showed the highest content of tyrosol equivalents (822.4 mg kg^−1^) when compared to the other maize samples.

**Table 3:** Semi-quantitative data for the different phenolic classes, as phenolic equivalents (±standard deviation) following UHPLC/QTOF profiling in each of the four maize genotypes.

Phenolic class	Phenolic equivalents (mg kg^−1^)			
	Agrister	Rostrato Rosso	Val di Non	Purple B73
Anthocyanins	205.39 ± 36.69	4399.42 ± 1862.37	752.54 ± 399.19	3167.94 ± 526.02
Flavones	16.52 ± 3.70	242.64 ± 87.97	40.68 ± 19.96	112.85 ± 14.85
Flavonols	17.86 ± 10.80	131.65 ± 59.63	33.73 ± 15.60	342.74 ± 66.88
Tyrosols	365.35 ± 58	822.35 ± 177.51	352.60 ± 78.40	464.15 ± 63.21
Hydroxycinnamic acids	979.64 ± 282.22	1149.68 ± 272.21	589.15 ± 262.26	1305.92 ± 480.18
Furofurans	24.02 ± 6.64	40.44 ± 15.74	16.65 ± 4.41	60.14 ± 29.31
Alkylphenols	42.21 ± 24.10	33.75 ± 7.03	53.42 ± 18.78	30.36 ± 4.02
Stilbenes	5.87 ± 1.77	2.41 ± 1.79	4.05 ± 3.34	1.39 ± 0.51

Subsequently, the OPLS-DA analysis was performed in order to better account for markers of the differences observed in phenolic profile. The predictive model clearly discriminated among maize cultivars (**Figure 4B**), showing that the pigmented genotypes, i.e., “Rostrato Rosso” and “Purple B73” samples possessed a completely differentiated phenolic profile when compared to the other samples, the latter clustering together onto the OPLS-DA hyperspace. The characteristics of the OPLS-DA model were excellent, with *R*^2^Y and *Q*^2^Y being 0.97 and 0.88, respectively. No outliers could be identified using Hotelling’s T2, whereas OPLS-DA over fitting could be excluded by both CV-ANOVA (correlation p 6.2 10^−17^) and permutation testing (**Supplementary Figure S1**). Afterwards, the variable’s importance in the OPLS-DA model was evaluated using the VIP analysis and exporting the VIP scores for each phenolic compound detected through the untargeted UHPLC-ESI/QTOF-MS approach. The VIP score summarizes the contribution that a variable makes to the OPLS-DA model. The phenolic compounds with the highest recorded VIP scores (>1.2) are reported in **Table 4**; the 42 phenolic compounds detected could be considered the most important and contributing variables in class discrimination. In line with the previously reported evaluations from unsupervised multivariate statistics and Volcano analysis, the most abundant phenolics identified by the VIP analysis could be ascribed to flavonoids (i.e., anthocyanins and flavones) and phenolic acids (above all, hydroxycinnamics), thus confirming that these two phenolic classes are the most explicative in determining the differences observed in phenolic profile.

**Table 4:** Compounds better discriminating between different maize genotypes, as selected by VIP analysis following OPLS-DA.

Phenolic class	Phenolic subclass	Compound	VIP score
Flavonoids	Anthocyanins	Pelargonidin 3-*O*-(6″-malonyl-glucoside)	1.34 ± 0.14
		Cyanidin 3-*O*-(6″-malonyl-glucoside)	1.33 ± 0.15
		Peonidin 3-*O*-(6″-malonyl-glucoside)	1.32 ± 0.17
		Malvidin 3-*O*-(6″-caffeoyl-glucoside)	1.32 ± 0.19
		Cyanidin 3-*O*-galactoside	1.30 ± 0.31
		Malvidin 3-*O*-glucoside	1.28 ± 0.37
		Cyanidin	1.22 ± 0.33
		Petunidin 3,5-*O*-diglucoside	1.20 ± 0.41
	Dihydroflavonols	Dihydromyricetin 3-*O*-rhamnoside	1.30 ± 0.31
		Dihydroquercetin	1.25 ± 0.29
	Flavanones	2 Hydroxy-eriodictyol	1.20 ± 0.34
	Flavones	Luteolin 7-*O*-diglucuronide	1.33 ± 0.18
		Chrysoeriol 7-*O*-(6″-malonyl-glucoside)	1.32 ± 0.19
		Apigenin 7-*O*-diglucuronide	1.32 ± 0.23
		6-Hydroxyluteolin 7-*O*-rhamnoside	1.30 ± 0.31
		Apigenin 7-*O*-apiosyl-glucoside	1.21 ± 0.24
	Flavonols	Kaempferol 3-*O*-glucosyl-rhamnosyl-galactoside	1.58 ± 0.24
		Jaceidin 4′-*O*-glucuronide	1.34 ± 0.11
		5,4′-Dihydroxy-3,3′-dimethoxy-6:7-methylenedioxyflavone 4′-*O*-glucuronide	1.33 ± 0.16
		Kaempferol 3-*O*-(2″-rhamnosyl-galactoside) 7-*O*-rhamnoside	1.32 ± 0.48
		Kaempferol 3,7,4′-*O*-triglucoside	1.29 ± 0.31
		Myricetin 3-*O*-rhamnoside	1.27 ± 0.19
		Kaempferol 3-*O*-xylosyl-rutinoside	1.26 ± 0.54
		Kaempferol	1.24 ± 0.34
		Quercetin 3-*O*-glucosyl-xyloside	1.22 ± 0.25
		Quercetin 3-*O*-arabinoside	1.20 ± 0.29
	Isoflavonoids	6″-*O*-Malonylgenistin	1.34 ± 0.10
		6″’-*O*-Acetylgenistin	1.20 ± 0.46
Phenolic acids	Hydroxycinnamics	24-Methylcholestanol ferulate	1.52 ± 0.43
		Isoferulic acid	1.34 ± 0.90
		1-Caffeoylquinic acid	1.30 ± 0.88
		3-Feruloylquinic acid	1.23 ± 0.30
		3-Sinapoylquinic acid	1.22 ± 0.59
		1,3-Dicaffeoylquinic acid	1.22 ± 0.19
	Hydroxyphenylacetics	Methoxyphenylacetic acid	1.37 ± 0.70
Others	Lignans	7-Hydroxysecoisolariciresinol	1.59 ± 0.17
		Episesaminol	1.44 ± 0.68
		Medioresinol	1.35 ± 0.23
		Episesamin	1.21 ± 0.32
	Alkylresorcinols	5-Tricosenylresorcinol	1.26 ± 0.90
		5-Nonadecenylresorcinol	1.20 ± 0.89
	Tyrosols	Hydroxytyrosol	1.35 ± 0.80

*Compounds are provided together with VIP scores ± standard error (measure of variable’s importance in the OPLS-DA model).*

### *In vitro* Antioxidant Capacity of the Maize Kernels

In this work, the *in vitro* antioxidant capacity of different maize genotypes was evaluated by means of two different methods, being the DPPH radical scavenging and the FRAP reducing power, since the aforementioned methods are based on different reaction kinetics, i.e., a hydrogen atom transfer (DPPH method) and a single electron transfer (FRAP). The results for antioxidant capacity are reported in **Table 5**. Remarkably, both assays provided essentially the same information, when compared to the cumulative abundances by UHPLC-ESI/QTOF-MS for each phenolic class equivalent. DPPH values were in the range 18.8 – 150.1 mg kg^−1^ GAE, whereas FRAP values were comprised between 4364.6 and 18616.2 mg kg^−1^ GAE. The FRAP results were consistent with the semi-quantitative values, recording the highest values in “Rostrato Rosso” followed by “Purple B73,” “Val di Non” and the Agrister yellow maize samples.

**Table 5:** *In vitro* antioxidant capacity values in the selected maize genotypes, as obtained through DPPH radical scavenging and FRAP reducing power assays.

	mg kg^−1^ gallic acid equivalents	
	DPPH	FRAP
Agrister	19.9 ± 7^a^	4364.6 ± 242.4^a^
Val di Non	21.4 ± 4.1^a^	5567.2 ± 258.6^a^
Purple B73	18.8 ± 1.5^a^	6139.1 ± 396.6^a^
Rostrato Rosso	150.1 ± 86.8^b^	18616.2 ± 6060^b^

*Within the same column, means followed by the same letter are not significantly different at P ≤ 0.05 according to Fisher’s test.*

### Response to Artificial Infection With *F. verticillioides*

**FIGURE 5:**
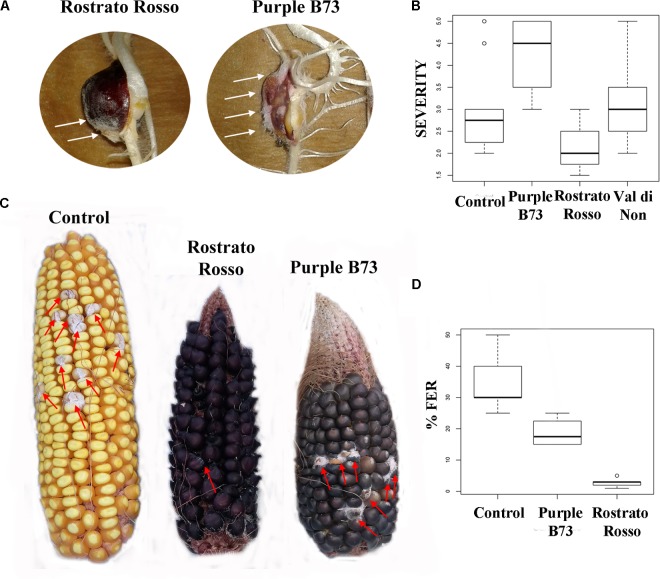
Pictures and box-plots of maize kernels artificially inoculated with *F. verticillioides*. Examples of treated seedling after RTA assay **(A)**; the same genotypes after ear inoculation in field **(C)**. Box plots representing severity after inoculation in RTA **(B)** and percentage of FER after field inoculation **(D)**.

The inbred line B73 was chosen as a yellow kernel reference because of its common use in association panels to screen for FER resistance (Zila et al., 2013). Previous literature on the concentration of beneficial phytochemicals in harvested grains of yellow maize highlighted a moderate variability of phenolic profile, mainly ascribable to a year × genotype interaction (Butts-Wilmsmeyer et al., 2017). Our UHPLC-ESI/QTOF-MS phenolic profiling of the B73 non-pigmented maize, followed by quantification as phenolic sub-classes, resulted in a substantially comparable profile, as compared to the Agrister genotype used as reference for antioxidant activity (**Supplementary Tables S2**, **S3**). The modest differences we observed between the two yellow maize samples could be likely ascribed to the above-mentioned year × genotype interaction. Nonetheless, the use of a commercial hybrid such as Agrister (i.e., the genotype used as reference in phenolic profiling) would not have been suitable for *in vitro* infection assays, because these seeds were commercialized as fungicide-coated kernels. All maize genotypes in the control RTA were free from *F. verticillioides* presence, with the exception of “Purple B73” and “Val di Non” (mean value of 1.25). After artificial inoculation different responses could be observed: “Rostrato Rosso” was the less infected with SEV_I means of 2.1 while “Val di Non” and “Purple B73” were the most favorable for fungal development (3.3 and 4.2 of mean values, respectively) (**Figures 5A,B**). Analysis of variance of SEV_I phenotypic values, performed according to the Kruskal–Wallis test, resulted in a *p*-value = 4.196 × 10^−9^. Therefore, samples were compared each other to shed light to significant variations between genotypes (Kruskal–Dunn *post hoc* test). “Purple B73” was significantly different with respect to the other pigmented and not pigmented varieties. Likewise, “Rostrato Rosso” was found to be significantly different from all other maize genotypes, while “Val di Non” response to infection was not different from that of the control. These results suggest that within colored maize cultivars different levels of resistance can be evidenced: “Rostrato Rosso” was the most resistant and “Purple B73” was the most susceptible (**Table 6** and **Figures 5A,B**).

**Table 6:** *P-values* after Kruskal–Dunn *post hoc* test on severity of infection phenotypic values following RTA inoculation with *F. verticillioides* (SEV_I) and *in field* inoculation (FER).

SEV_I	B73	Purple B73	Val di Non	FER	B73	Purple B73
Purple B73	0.002^∗^	#	#	Purple B73	0.05^∗^	#
Val di Non	0.2919	0.0483^∗^	#	Rostrato Rosso	<0.01^∗^	0.01^∗^
Rostrato Rosso	0.0136^∗^	1 × 10^−9∗^	0.0008^∗^			

*Significant comparisons are marked by an asterisk.*

### Field Evaluation for FER Resistance

Field-grown maize genotypes were visually scored for FER severity following artificial inoculation, and variation between accessions is shown in **Figures 5C,D**. Because ears of the cultivar “Val di Non” were poorly pollinated, they were not used for *Fusarium* infection analysis in order to prevent an incorrect FER evaluation (Walker and White, 2001).

ANOVA after Welch test resulted in significant differences (*p* = 0.00102) and thus the Games–Howell *post hoc* test was applied to check for homogenous classes (**Table 6**). All the genotypes were significantly different each other, with the highest significance values considering comparisons to “Rostrato Rosso” (**Table 6**).

## Discussion

Pigmented maize genotypes were characterized by different contents in anthocyanins, and in other not pigmented phenolic compounds. Even though anthocyanins were the highest in “Rostrato Rosso” and “Purple B73,” phenolic acids were the second class in abundance, having the highest content in “Rostrato Rosso,” “Purple B73” and control. Tyrosols and flavones were highest in “Rostrato Rosso,” whereas “Purple B73” showed the highest flavonols content. Therefore, the difference in phenolic profile among the considered genotypes was far beyond what observed at phenotype level. With this regard, an untargeted metabolomic profiling approach appears to be more appropriate in describing the actual phenolic profile from a holistic perspective. Indeed, VIP analysis from OPLS-DA multivariate statistics highlighted that compounds belonging to flavonoids such as flavones and flavonols, rather than hydroxycinnamic acids, were also responsible of differences in phenolic profile between cultivars, together with anthocyanins. Previous literature on phenolics in pigmented maize mainly focused on anthocyanins profile, highlighting as the malonyl and succinyl derivatives are the predominant one (Carmelo-Méndez et al., 2016). The results obtained confirm the importance of acylated anthocyanins, whereas total anthocyanins in our samples were in the same range of other pigmented maize (Lopez-Martinez et al., 2009) and one order higher than values reported for Mexican blue maize (Mora-Rochín et al., 2016). However, to the best of our knowledge, no comparative information has been reported regarding the phenolic content of other classes of phenolic compounds.

A confirmation on the relevance of colorless phenolics also in pigmented maize can be gained looking at Pearson’s correlation values (**Table 7**) between the *in vitro* antioxidant capacity and the phenolic classes content. Tyrosols and anthocyanins were highly correlated to FRAP reducing power (0.87 and 0.86, respectively, *p* < 0.01) whereas DPPH radical scavenging capacity correlated with flavones, tyrosols and anthocyanins (0.85, 0.80, and 0.70, respectively, *p* < 0.01). A significant but weak correlation was observed between DPPH and FRAP values (0.47, *p* < 0.01); this result is not surprising, considering that the different antioxidant tests rely on different mechanisms and should be better considered as complementary rather than alternative (Przygodzka et al., 2014). Indeed, the *in vitro* antioxidant capacity should not be determined based on a single antioxidant test model (Alam et al., 2013). Interestingly, the spread of DPPH values was much narrower than FRAP reducing power values, suggesting that DPPH assay might be less informative for the description of antioxidant capacity in the maize samples analyzed. Nonetheless, our results regarding antioxidant capacity values were consistent with previous studies. In particular, the antioxidant capacity of pigmented maize samples and by-products has been previously studied (Bello-Pérez et al., 2015), with anthocyanins giving the most important contribution to the FRAP, DPPH, and ABTS recorded values (Bello-Pérez et al., 2015; Carmelo-Méndez et al., 2017). However, most of the available studies in literature investigated the use pigmented maize varieties as potential ingredient for the development of functional foods, but focusing the attention above all on blue maize.

**Table 7:** Two-tails Pearson correlation between *in vitro* antioxidant capacity assays and content of the main phenolic classes.

	FRAP	DPPH	Anthocyanins	Flavonols	Flavones	Tyrosols	Hydroxycinnamics
FRAP	–	0.47^∗^	0.86^∗∗^	n.s.	0.75^∗∗^	0.87^∗∗^	n.s.
DPPH	0.47^∗^	–	0.70^∗^	n.s.	0.85^∗∗^	0.80^∗∗^	n.s.
Anthocyanins	0.86^∗∗^	0.70^∗∗^	–	n.s.	0.96^∗∗^	0.97^∗∗^	0.58^∗∗^
Flavonols	n.s.	n.s.	n.s.	–	n.s.	n.s.	0.79^∗∗^
Flavones	0.75^∗∗^	0.85^∗∗^	0.96^∗∗^	n.s.	–	0.97^∗∗^	0.47^∗^
Tyrosols	0.87^∗∗^	0.80^∗∗^	0.97^∗∗^	n.s.	0.97^∗∗^	–	0.48^∗^
Hydroxycinnamics	n.s.	n.s.	0.58^∗∗^	0.79^∗∗^	0.47^∗^	0.48^∗^	–
RTA infection	−0.60^∗∗^	−0.57^∗∗^	−0.52^∗^	n.s.	−0.57^∗∗^	−0.61^∗∗^	n.s.
Field infection	−0.64^∗∗^	−0.54^∗^	−0.80^∗∗^	−0.46^∗^	−0.73^∗∗^	−0.78^∗∗^	−0.79^∗∗^

*Significant correlations are indicated by asterisks (^∗^p < 0.05, ^∗∗^p < 0.01).*

Moving toward the involvement of phenolic profiles in resistance toward *Fusarium* infection, interesting differences could be pointed out from both RTA and field inoculation experiments. The genotype “Rostrato Rosso” was the most resistant after *F. verticillioides* infection, both *in vitro* and in field conditions. Interestingly, this cultivar was featured by a significantly higher phenolic content. “Purple B73” presented a more severe infection following *in vitro* and field inoculation with *Fusarium*, as compared to pigmented genotypes.

With this regard, it is important to highlight that plants use a complex and interconnected defense system against pests and pathogens; the production of low molecular weight secondary metabolites having antimicrobial activity, collectively known as phytoalexins, is part of these defense mechanisms (Ahuja et al., 2012). Previous literature reported that the kernel color can be associated to *Fusarium* resistance and that the accumulation of flavonoids pigments in kernel is able to reduce the accumulation of fumonisin B1 (Pilu et al., 2011). Nonetheless, the localization of pigments in maize kernel might have a prominent role in the actual degree of resistance to *Fusarium* infection. Recently, it was reported that flavonoids can inhibit fumonisin accumulation in *Fusarium*-inoculated maize ears, even though pigmented pericarp alone was ineffective in preventing the accumulation of the mycotoxin (Venturini et al., 2016).

## Conclusion

The different pigmented maize cultivars selected in this work appear to be interesting both in terms of phenolic profile and antioxidant capacity. Therefore, the findings support further studies in order to formulate food matrices enriched in phenolic compounds, and thus with beneficial health-promoting effects.

Given the fact that the highest resistance to *Fusarium* infection was observed in the cultivar having the highest phenolic content, further studies should be made to confirm the association between specific phenolic compounds or classes of phenolics, and fungal resistance. Undeniably, pigmented maize cultivars could be important both in finding breeding strategies in the framework of sustainable agriculture as well as in developing foods/food ingredients with higher nutraceutical value.

## Author Contributions

All authors contributed to revise work critically, gave the final approval of the version to be published and agreed on all aspects of the work. In particular, AM designed the study, in cooperation with JB and LL. JB, LS, and MB carried out the field experiments and the artificial infections, contributed to interpretation of data, drafted and revised the manuscript. LL and GR developed the mass spectrometric method, performed statistics, and drafted the manuscript. AL, MB, CC, and GDP drafted and critically revised the manuscript.

## Conflict of Interest Statement

The authors declare that the research was conducted in the absence of any commercial or financial relationships that could be construed as a potential conflict of interest.
