# Supplementary figures and images for: Phenolic Profile and Susceptibility to Fusarium Infection of Pigmented Maize Cultivars
Source: Front Plant Sci. 2018 Aug 14;9:1189. doi: 10.3389/fpls.2018.01189 (PMC6102558; doi:10.3389/fpls.2018.01189)

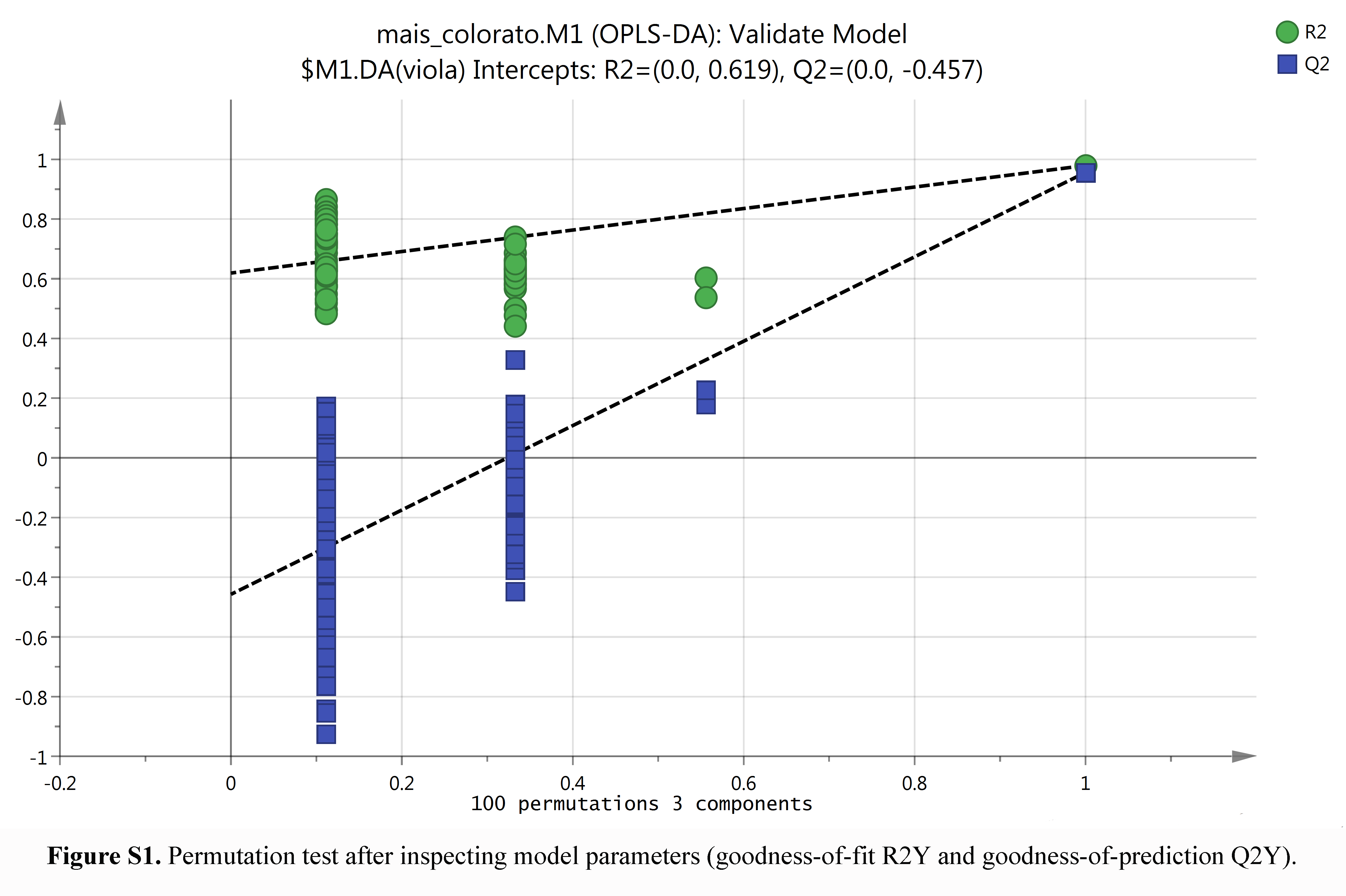

Supplement: Supplementary file 1 [file Image_1.TIF]
