# Supplementary material for: Phenolic Profile and Susceptibility to Fusarium Infection of Pigmented Maize Cultivars
Source: Front Plant Sci. 2018 Aug 14;9:1189. doi: 10.3389/fpls.2018.01189 (PMC6102558; doi:10.3389/fpls.2018.01189)
Supplement: Supplementary file 2 [file Table_1.DOCX]

**Table S1.** **List of compounds that are differentially accumulated comparing each colored variety vs control.** a) all compounds; b) only up regulated compounds

**a)**

| **8 compounds included exclusively in "Val di Non":** | | |
| --- | --- | --- |
| m-Coumaric acid |  | Hydroxycinnamic acids |
| Syringaresinol |  | Lignans |
| p-Coumaric acid 4-O-glucoside |  | Hydroxycinnamic acids |
| Coumarin |  | Hydroxycoumarins |
| Oleoside 11-methylester |  | Tyrosols |
| Hydroxycaffeic acid |  | Hydroxycinnamic acids |
| Mellein |  | Hydroxycoumarins |
| epsilon-carotene |  | Carotenoids |
|  |  |  |
| **22 compounds included exclusively in "Rostrato Rosso":** | | |
| (-)-Epigallocatechin 3-O-gallate | Flavanols |  |
| 1,2-Disinapoylgentiobiose |  | Hydroxycinnamic acids |
| 4-Hydroxybenzoic acid 4-O-glucoside |  | Hydroxybenzoic acids |
| 4-Vinylsyringol |  | Alkylmethoxyphenols |
| Medioresinol |  | Lignans |
| Methoxyphenylacetic acid |  | Hydroxyphenylacetic acids |
| Pelargonidin 3-O-glucosyl-rutinoside | Anthocyanins |  |
| Phlorin |  | Other polyphenols |
| Pyrogallol |  | Other polyphenols |
| Sinapaldehyde |  | Hydroxycinnamaldehydes |
| Sinapine |  | Hydroxycinnamic acids |
| 5-5'-Dehydrodiferulic acid |  | Hydroxycinnamic acids |
| Biochanin A | Isoflavonoids |  |
| Rhoifolin | Flavones |  |
| Cyanidin 3,5-O-diglucoside | Anthocyanins |  |
| Cyanidin 3-O-sambubioside | Anthocyanins |  |
| Delphinidin 3-O-xyloside | Anthocyanins |  |
| Diosmin | Flavones |  |
| Isorhamnetin 3-O-rutinoside | Flavonols |  |
| Kaempferol 3,7-O-diglucoside | Flavonols |  |
| Malvidin 3-O-(6''-acetyl-galactoside) | Anthocyanins |  |
| Pelargonidin 3-O-sophoroside | Anthocyanins |  |
|  |  |  |
| **17 compounds included exclusively in "Purple B73":** |  |  |
| 5,6-Dihydroxy-7,8,3',4'-tetramethoxyflavone | Flavones |  |
| Dihydrocaffeic acid |  | Hydroxyphenylpropanoic acids |
| Peonidin | Anthocyanins |  |
| 6''-O-Malonylglycitin | Isoflavonoids |  |
| Arbutin |  | Other polyphenols |
| Chrysoeriol 7-O-(6''-malonyl-apiosyl-glucoside) | Flavones |  |
| 5-Heneicosenylresorcinol |  | Alkylphenols |
| Kaempferol 3-O-glucosyl-rhamnosyl-galactoside | Flavonols |  |
| Oleuropein |  | Tyrosols |
| 24-Methylcholestanol ferulate |  | Hydroxycinnamic acids |
| Episesaminol |  | Lignans |
| Petunidin 3-O-galactoside | Anthocyanins |  |
| Matairesinol |  | Lignans |
| 6-Hydroxyluteolin 7-O-rhamnoside | Flavones |  |
| Kaempferol 3-O-xylosyl-rutinoside | Flavonols |  |
| Cyanidin 3-O-(6''-malonyl-3''-glucosyl-glucoside) | Anthocyanins |  |
| Kaempferol 3,7,4'-O-triglucoside | Flavonols |  |
|  |  |  |
| **30 common compounds in "Val di Non", "Rostrato Rosso" and "Purple B73":** | | |
| Capsanthin |  | Carotenoids |
| Isoferulic acid |  | Hydroxycinnamic acids |
| Quercetin 4'-O-glucoside | Flavonols |  |
| 7-Hydroxysecoisolariciresinol |  | Lignans |
| Cyanidin 3-O-glucosyl-rutinoside | Anthocyanins |  |
| Jaceosidin | Flavones |  |
| Carnosic acid |  | Phenolic terpenes |
| Juglone |  | Naphtoquinones |
| Phloridzin | Dihydrochalcones | |
| Theaflavin | Flavanols |  |
| Apigenin 6-C-glucoside | Flavones |  |
| Pelargonidin 3-O-galactoside | Anthocyanins |  |
| Chrysoeriol 7-O-(6''-malonyl-glucoside) | Flavones |  |
| Pinocembrin | Flavanones |  |
| Apigenin 7-O-(6''-malonyl-apiosyl-glucoside) | Flavones |  |
| Homoveratric acid |  | Hydroxyphenylacetic acids |
| Pelargonidin 3-O-arabinoside | Anthocyanins |  |
| 5-Pentadecylresorcinol |  | Alkylphenols |
| Apigenin 7-O-apiosyl-glucoside | Flavones |  |
| 3,4-DHPEA-EA |  | Tyrosols |
| Peonidin 3-O-(6''-malonyl-glucoside) | Anthocyanins |  |
| Apigenin 6,8-di-C-glucoside | Flavones |  |
| Cyanidin 3-O-rutinoside | Anthocyanins |  |
| Sakuranetin | Flavanones |  |
| (+)-Gallocatechin | Flavanols |  |
| Myricetin 3-O-rhamnoside | Flavonols |  |
| Gardenin B | Flavones |  |
| p-Coumaroyl tyrosine |  | Hydroxycinnamic acids |
| Malvidin 3-O-(6''-caffeoyl-glucoside) | Anthocyanins |  |
| Gallic acid |  | Hydroxybenzoic acids |

**b)**

| **compound included exclusively in "Val di Non":** | | |
| --- | --- | --- |
| epsilon-carotene |  | Carotenoids |
|  |  |  |
| **13 compounds included exclusively in "Rostrato Rosso":** | | |
| Malvidin 3-O-(6''-acetyl-galactoside) | Anthocyanins |  |
| Cyanidin 3,5-O-diglucoside | Anthocyanins |  |
| Rhoifolin | Flavones |  |
| 5-5'-Dehydrodiferulic acid |  | Hydroxycinnamic acids |
| Biochanin A | Isoflavonoids |  |
| Neoeriocitrin | Flavanones |  |
| Diosmin | Flavones |  |
| Delphinidin 3-O-xyloside | Anthocyanins |  |
| Cyanidin 3-O-sambubioside | Anthocyanins |  |
| Kaempferol 3,7-O-diglucoside | Flavonols |  |
| Pelargonidin 3-O-sophoroside | Anthocyanins |  |
| Peonidin 3-O-rutinoside | Anthocyanins |  |
| Isorhamnetin 3-O-rutinoside | Flavonols |  |
|  |  |  |
| **8 compounds included exclusively in "Purple B73":** | | |
| Dihydromyricetin 3-O-rhamnoside | Dihydroflavonols |  |
| Pelargonidin 3-O-(6''-malonyl-glucoside) | Anthocyanins |  |
| 6-Hydroxyluteolin 7-O-rhamnoside | Flavones |  |
| Kaempferol 3-O-xylosyl-rutinoside | Flavonols |  |
| Jaceidin 4'-O-glucuronide | Flavonols |  |
| 6''-O-Malonylgenistin | Flavonols |  |
| Cyanidin 3-O-(6''-malonyl-3''-glucosyl-glucoside) | Flavonols |  |
| Kaempferol 3,7,4'-O-triglucoside | Flavonols |  |
|  |  |  |
| **20 common compounds in "Val di Non", "Rostrato Rosso" and "Purple B73":** | | |
| Juglone |  | Naphtoquinones |
| Phloridzin | Dihydrochalcones |  |
| Theaflavin | Flavanols |  |
| Apigenin 6-C-glucoside | Flavones |  |
| Pelargonidin 3-O-galactoside | Anthocyanins |  |
| Chrysoeriol 7-O-(6''-malonyl-glucoside) | Flavones |  |
| Pinocembrin | Flavanones |  |
| Apigenin 7-O-(6''-malonyl-apiosyl-glucoside) | Flavones |  |
| Homoveratric acid |  | Hydroxyphenylacetic acids |
| Pelargonidin 3-O-arabinoside | Anthocyanins |  |
| 5-Pentadecylresorcinol |  | Alkylphenols |
| 3,4-DHPEA-EA |  | Tyrosols |
| Peonidin 3-O-(6''-malonyl-glucoside) | Anthocyanins |  |
| Sakuranetin | Flavanones |  |
| (+)-Gallocatechin | Flavanols |  |
| Myricetin 3-O-rhamnoside | Flavonols |  |
| Gardenin B | Flavones |  |
| p-Coumaroyl tyrosine |  | Hydroxycinnamic acids |
| Malvidin 3-O-(6''-caffeoyl-glucoside) | Anthocyanins |  |
| Gallic acid |  | Hydroxybenzoic acids |
